# LncRNA HOXA-AS3 confers cisplatin resistance by interacting with HOXA3 in non-small-cell lung carcinoma cells

**DOI:** 10.1038/s41389-019-0170-y

**Published:** 2019-10-15

**Authors:** Shuang Lin, Rui Zhang, Xiaoxia An, Zhoubin Li, Cheng Fang, Bo Pan, Wei Chen, Guodong Xu, Weili Han

**Affiliations:** 1 Department of Lung Transplantation, Department of Thoracic Surgery, the First Affiliated Hospital, College of Medicine, Zhejiang University, Hangzhou, Zhejiang China; 2 Department of Internal medicine, Hangzhou Wuyunshan Sanatorium, the Affiliated Hangzhou First People’s Hospital, College of Medicine, Zhejiang University, Hangzhou, Zhejiang China; 3 Department of Anesthesiology, the First Affiliated Hospital, College of Medicine, Zhejiang University, Hangzhou, Zhejiang China; 4 Cancer Institute of Integrated traditional Chinese and Western Medicine, Key laboratory of cancer prevention and therapy combining traditional Chinese and Western Medicine, Zhejiang Academy of Traditional Chinese Medicine, Hangzhou, Zhejiang 310012 China; 5 Department of Cardiovascular Surgery, The Affiliated Hospital, Ningbo Medical Center Lihuili Hospital, Ningbo University, Ningbo, Zhejiang 315041 China

**Keywords:** Cell biology, Molecular biology

## Abstract

Many studies have indicated that the aberrant expression of long noncoding RNAs (lncRNAs) is responsible for drug resistance, which represents a substantial obstacle for cancer therapy. In the present study, we aimed to investigate the role of the lncRNA HOXA-AS3 in drug resistance and elucidate its underlying mechanisms in non-small-cell lung carcinoma (NSCLC) cells. The role of HOXA-AS3 in drug resistance was demonstrated by the cell counting kit-8 assay (CCK-8), ethynyldeoxyuridine (EDU) assay, and flow cytometry analysis. Tumor xenografts in nude mice were established to evaluate the antitumor effects of HOXA-AS3 knockdown in vivo. Western blotting and quantitative real-time PCR were used to evaluate protein and RNA expression. RNA pull-down assays, mass spectrometry, and RNA immunoprecipitation were performed to confirm the molecular mechanism of HOXA-AS3 in the cisplatin resistance of NSCLC cells. We found that HOXA-AS3 levels increased with cisplatin treatment and knockdown of HOXA-AS3 enhance the efficacy of cisplatin in vitro and in vivo. Mechanistic investigations showed that HOXA-AS3 conferred cisplatin resistance by down-regulating homeobox A3 (HOXA3) expression. Moreover, HOXA-AS3 was demonstrated to interact with both the mRNA and protein forms of HOXA3. In addition, HOXA3 knockdown increased cisplatin resistance and induced epithelial-mesenchymal transition (EMT). Taken together, our findings suggested that additional research into HOXA-AS3 might provide a better understanding of the mechanisms of drug resistance and promote the development of a novel and efficient strategy to treat NSCLC.

## Introduction

Lung cancer is one of the most common cancers and is the predominant cause of cancer-related mortality worldwide^1^. Non-small-cell lung carcinoma (NSCLC) accounts for approximately 85% of lung cancer cases. Although the prognosis of lung cancer has improved because of early diagnosis, radical surgery, and the emergence of new adjuvant chemotherapy regimens and targeted biological agents, the prognosis of NSCLC remains poor^2^. One of the main reasons for this is anticancer drug resistance. Among anticancer drugs, the development of cisplatin represents a successful milestone in the history of NSCLC therapy. Cisplatin forms intrastrand crosslinks in DNA, which disturbs DNA replication and causes DNA damage, eventually inducing necrosis or apoptosis. Cisplatin-based chemotherapy is widely used to treat NSCLC and has been shown to improve patient survival rates^3^. However, drug resistance is a complex process and often occurs during cisplatin treatment. Moreover, the mechanism underlying cisplatin resistance has remained elusive.

Long noncoding RNAs (lncRNAs) are transcripts of more than 200 nucleotides without obvious protein-coding functions. In recent years, abnormal lncRNA expression has been found in many types of tumors, playing roles in regulating cancer cell proliferation, differentiation, invasion, and metastasis^4,5^. Importantly, lncRNAs are considered key regulators in drug resistance, and may also act as promising prognostic and therapeutic targets. Therefore, lncRNA functions in cancer have become an area of extensive research^6,7^. Despite recent studies showing that lncRNAs could confer chemo-resistance in cancer cells by improving DNA repair, cellular apoptosis, epithelial-mesenchymal transition (EMT), and altering drug metabolism and membrane efflux^8^, the molecular mechanisms underlying the action of lncRNAs in these cancer cell functions remain largely elusive. A better understanding of the molecular mechanisms of lncRNAs in drug resistance may lead to the development of more effective cancer treatments.

EMT is a process by which epithelial cells lose apical-basal polarity and cell-cell adhesion and migrate to invasive mesenchymal cells. Recently, EMT has received increasing attention for its role in cancer drug resistance^9,10^. Many EMT-related signaling pathways are involved in drug resistance in cancer cells. For example, long non-coding RNA NONHSAT101069 promotes epirubicin resistance through NONHSAT101069/miR-129-5p/Twist1 axis^11^. The PAX6-ZEB2 axis promotes cisplatin resistance in non-small cell lung cancer through PI3K/AKT signaling^12^. However, whether EMT was involved in lncRNA-mediated drug resistance in NSCLC is still unclear.

In the present study, we aimed to investigate the role and underlying mechanisms of lncRNAs in mediating cisplatin resistance in NSCLC, by focusing on an lncRNA with high expression in NSCLC, HOXA-AS3. Our findings suggested that HOXA-AS3 may be a novel molecular target to treat patients with NSCLC.

## Results

### Responses of NSCLC cell lines and HOXA-AS3 levels to cisplatin treatment

**Fig. 1:**
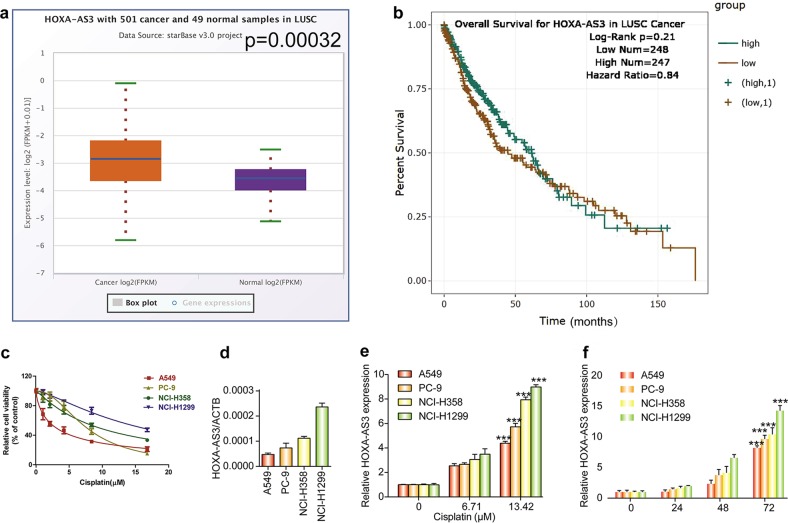
NSCLC cells display different responses to cisplatin treatment and HOXA-AS3 expression increases with cisplatin treatment. **a** The StarBase v 3.0 project was used to analyze the level of HOXA-AS3 in 501 cancer and 49 normal samples in LUSC. **b** Survival curve used to determine the level of HOXA-AS3 in LUSC. **c** CCK-8 assay of the viability of NSCLC cell lines treated with increasing concentrations (0, 1.049, 2.097, 4.195, 8.389, 16.779 μM) of cisplatin for 48 h. **d** qPCR analyses of the HOXA-AS3 basal level in four NSCLC cell lines under the IC_50_ of cisplatin for 48 h. **e** qPCR analyses of the HOXA-AS3 expression in NSCLC cell lines with increasing concentrations of cisplatin for 48 h. **f** qPCR analyses of the HOXA-AS3 expression of NSCLC cell lines under the IC_50_ of cisplatin at different times. (*p < 0.05, **p < 0.01, ***p < 0.001)

To investigate the potential role of HOXA-AS3 in NSCLC, we first used the StarBasev.3 project to analyze the level of HOXA-AS3 in LUSC (lung squamous cell carcinoma). As expected, the level of HOXA-AS3 was higher in 501 LUSC samples than in 49 normal samples (Fig. 1a). The results of the survival curve showed that there was no statistical difference in survival of patients with LUSC cancer with high or low HOXA-AS3 expression (Fig. 1b). To determine the role of HOXA-AS3 in mediating resistance to chemotherapy, four NSCLC cell lines (A549, PC-9, NCI-H358, and NCI-H1299) were used to investigate the effect of cisplatin treatment. The results showed that the viability of each cell line was reduced by cisplatin in a dose-dependent manner (Fig. 1c). The basal level of HOXA-AS3 expression was lowest in A549 cells, followed by PC-9, NCI-H358, and NCI-H1299 cells, and resistance to cisplatin appeared to be greater in cells expressing higher levels of HOXA-AS3 (Fig. 1d). Furthermore, we determined the IC_50_ concentration of cisplatin in each cell line and found the same pattern, with an IC_50_ of 3.027 (2.730 to 3.323) μM in A549, 7.205 (8.087 to 8.735) μM in PC-9, 9.51 (8.735 to 10.208) μM in NCI-H358, and 4.760 (14.923 to 17.023) μM in NCI-H1299 cells (Supplementary Table 1.1). Next, we assessed the expression levels of HOXA-AS3 following cisplatin treatment. Significant upregulation of HOXA-AS3 was observed in NSCLC cells following treatment with cisplatin, in a dose-dependent manner (Fig. 1e). In addition, HOXA-AS3 levels also increased significantly in a time-dependent manner following cisplatin treatment (Fig. 1f). These results suggested that HOXA-AS3 expression increased with cisplatin treatment in a dose- and time-dependent manner in NSCLC cells; therefore, HOXA-AS3 might be useful as a biomarker to predict cisplatin resistance.

### HOXA-AS3 confers cisplatin resistance in NSCLC cells

**Fig. 2:**
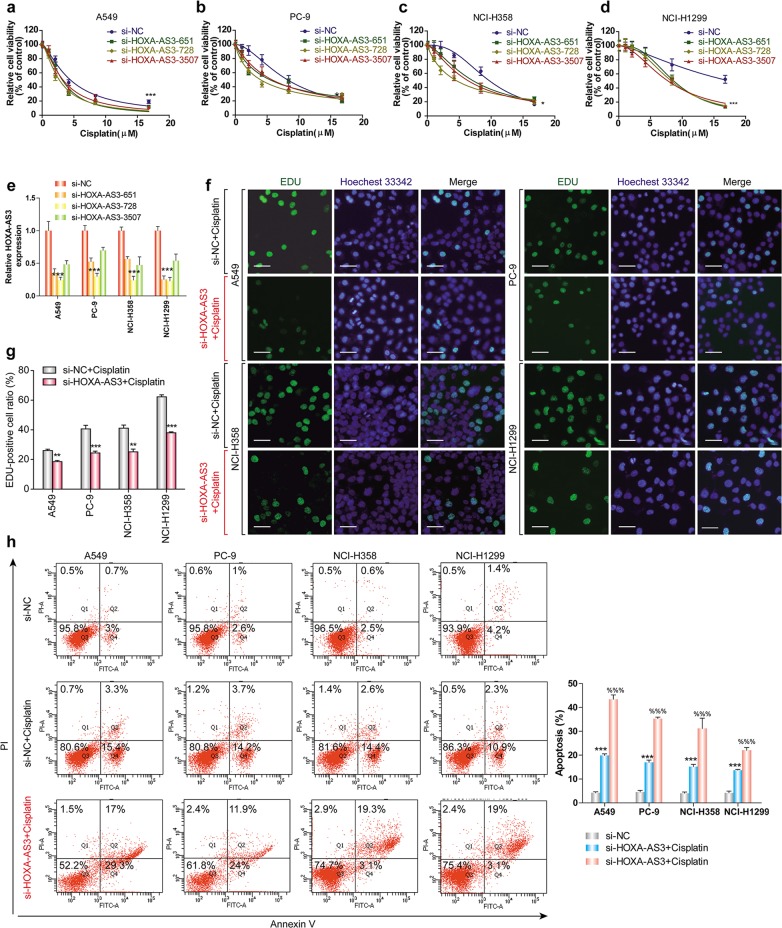
HOXA-AS3 confers cisplatin resistance in NSCLC cells. **a**–**d** CCK-8 assays of the viability of NSCLC cell lines following cisplatin treatment (0, 1.049, 2.097, 4.195, 8.389, 16.779 μM) under HOXA-AS3 suppression for 48 h. **e** qPCR analyses of HOXA-AS3 expression following its suppression. **f**, **g** EDU assay of the proliferation rate of NSCLC cell lines under the IC_50_ of cisplatin with HOXA-AS3 suppression for 48 h. All scale bars indicate 50 μm (Green, EDU staining of dividing cells; blue, Hoechest 33342staining of nuclei). **h** Flow cytometry detection of the apoptosis rate of NSCLC cell lines under the IC_50_ of cisplatin with HOXA-AS3 suppression for 48 h. (*p < 0.05, **p < 0.01, ***p < 0.001)

Considering that HOXA-AS3 expression increased with cisplatin treatment of NSCLC cell lines, we wondered whether suppressing HOXA-AS3 expression would increase the effect of cisplatin in the cell lines. We knocked down HOXA-AS3 expression using siRNAs and assessed NSCLC cell viability following cisplatin treatment. CCK-8 assays showed that knockdown of HOXA-AS3 resulted in a significant dose-dependent reduction in the viability of NSCLC cells in response to cisplatin treatment as compared with that in the control (Fig. 2a–e). After knockdown of HOXA-AS3, the IC_50_ values of cisplatin were also significantly reduced in each NSCLC cell line (Supplementary Table 1.2). EDU staining assays also revealed that knockdown of HOXA-AS3 decreased NSCLC cell proliferation following cisplatin treatment for 48 h, as compared with that in the control cells (Fig. 2f, g). Refractoriness to cisplatin-induced apoptosis is one of the main characteristics of chemotherapy resistance; therefore, the effect of HOXA-AS3 on apoptosis was examined. Flow cytometry analysis showed that knockdown of HOXA-AS3 led to a significant increase in NSCLC cell apoptosis following cisplatin treatment (Fig. 2h). To further investigate the biological function of HOXA-AS3 in cisplatin resistance, we overexpressed HOXA-AS3 in the four NSCLC cell lines through transfection with a HOXA-AS3-encoding vector. When HOXA-AS3 was overexpressed (Fig. S1e), cisplatin treatment had no effect on cell viability compared with that in the controls (Fig. S1a–d). The IC_50_ value of cisplatin in each NSCLC cell line was also unaffected (Supplementary Table 1.3). Taken together, these results suggested that HOXA-AS3 mayfunction as a cis-acting RNA that needs to function at specific locations (such as on some specific chromatin regions), to confer cisplatin resistance in vitro.

### HOXA-AS3 knockdown increases the expression of E-cadherin and decrease Vimentin expression

**Fig. 3:**
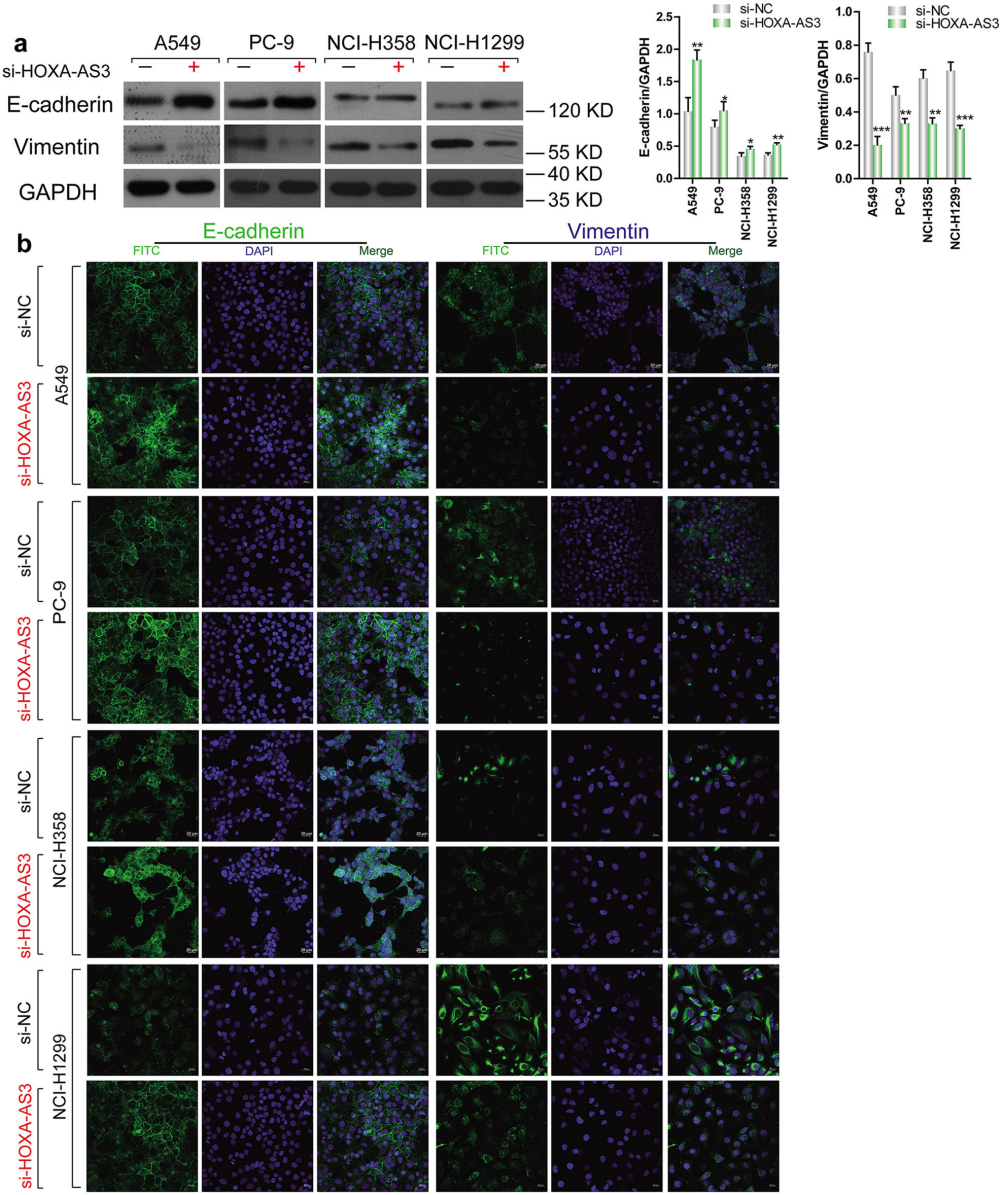
HOXA3-AS3 knockdown increases the expression of E-cadherin and decreases Vimentin expression. **a** Western blotting detection of E-cadherin and Vimentin levels following HOXA-AS3 knockdown or with wild-type HOXA-AS3 expression for 48 h. **b** Immunofluorescence was used to examine the expression of E-cadherin and Vimentinfor 48 h. (*p < 0.05, **p < 0.01, ***p < 0.001)

To determine the effect of HOXA-AS3 on the expression of E-cadherin and Vimentin, we used western blotting and immunofluorescenceto detect E-cadherin and Vimentin expression, respectively. The results showed that after transfection with the HOXA-AS3 siRNA, the level of E-cadherin was upregulated, while Vimentin was downregulated compared with the NC siRNA in NSCLC (Fig. 3a). Immunofluorescence also confirmed this results (Fig. 3b).

### HOXA-AS3 interacts with HOXA3 mRNA and protein

**Fig. 4:**
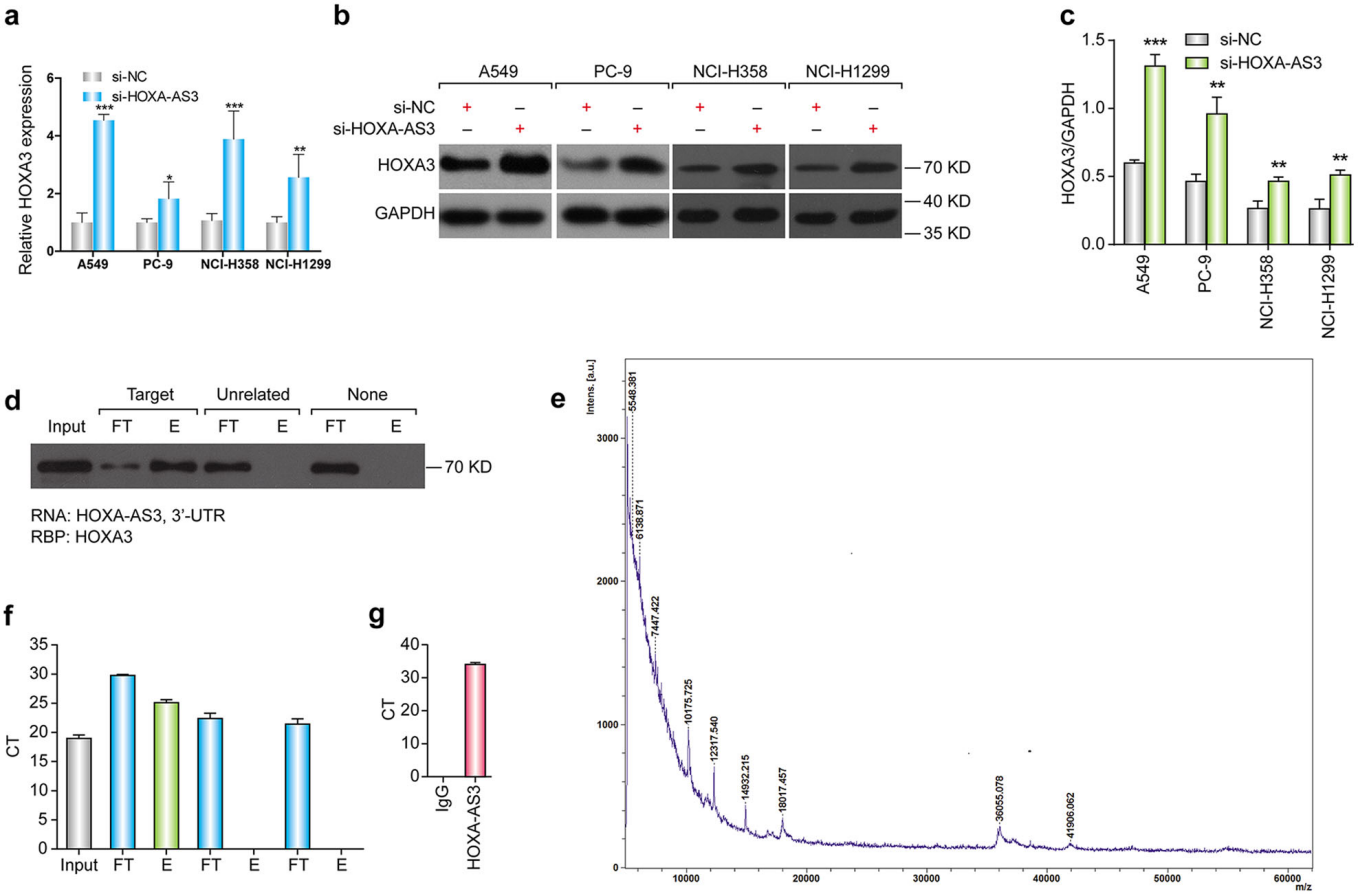
HOXA-AS3 interacted with HOXA3. **a** qPCR analyses of the mRNA expression of HOXA3 following HOXA-AS3 suppression for 48 h. **b**, **c** Western blotting detection of the protein levels of HOXA3 following HOXA-AS3 suppression for 48 h. **d** RNA pull-down assay and western blotting detection of HOXA-AS3 binding to the HOXA3 protein. (L lysate load, FT flow-through; E eluate). **e** Mass spectrum detection of HOXA-AS3 binding to the HOXA3 protein. **f** RNA pull-down assay and qPCR detection of HOXA-AS3 binding to the mRNA of HOXA3. (L lysate load, FT flow-through, E eluate). **g** RNA immunoprecipitation detection of the enrichment of HOXA-AS3 using an anti-HOXA3 antibody. (*p < 0.05, **p < 0.01, ***p < 0.001)

To determine how HOXA-AS3 affects the drug resistance of NSCLC, we conducted bioinformatics analysis to identify potential downstream target genes. Querying the UCSC genome browser (http://genome.ucsc.edu/) and the NCBI database (http://blast.ncbi.nlm.nih.gov/) showed that HOXA-AS3 was predicted to target the HOXA3 gene (encoding homeobox A3), raising the possibility that HOXA-AS3 affects the expression of HOXA3. We performed qPCR and western blotting analyses to confirm this hypothesis. The results showed that knockdown of HOXA-AS3 upregulated HOXA3 expression at both the mRNA and protein levels in all four NSCLC cell lines (Fig. 4a–c).

As noncoding transcripts, lncRNAs exert their functions by modulating the mRNA and protein expression of related target genes. We searched for potential proteins partners of HOXA-AS3 using RPISeq (http://pridb.gdcb.iastate.edu/RPISeq/index.html), a free online tool to predict RNA-protein interactions. Based on two classification algorithms [random forest (RF) and support vector machine (SVM)], the HOXA3 protein was predicted to bind to HOXA-AS3. We confirmed the interaction between HOXA-AS3 and the HOXA3 protein experimentally using an RNA pull-down assay and western blotting (Fig. 4d). Mass spectrometry also confirmed that the specific band in HOXA-AS3 samples was HOXA3 (Fig. 4e). We also examined the RNAs that may be bound by HOXA3-AS directly. Interestingly, the RNA pull-down assay and qPCR showed that HOXA-AS3 was capable of interacting with the HOXA3 mRNA (Fig. 4f). Additionally, we performed RNA immunoprecipitation (RIP) using antibodies against HOXA3 to examine if HOXA-AS3 was enriched using qPCR and found that HOXA-AS3 was indeed enriched in the HOXA3 RIP samples (Fig. 4g). Taken together, these results confirm that HOXA-AS3 interacts with the HOXA3 mRNA and protein in NSCLC cells.

### HOXA3 knockdown increases cisplatin resistance and induces EMT

**Fig. 5:**
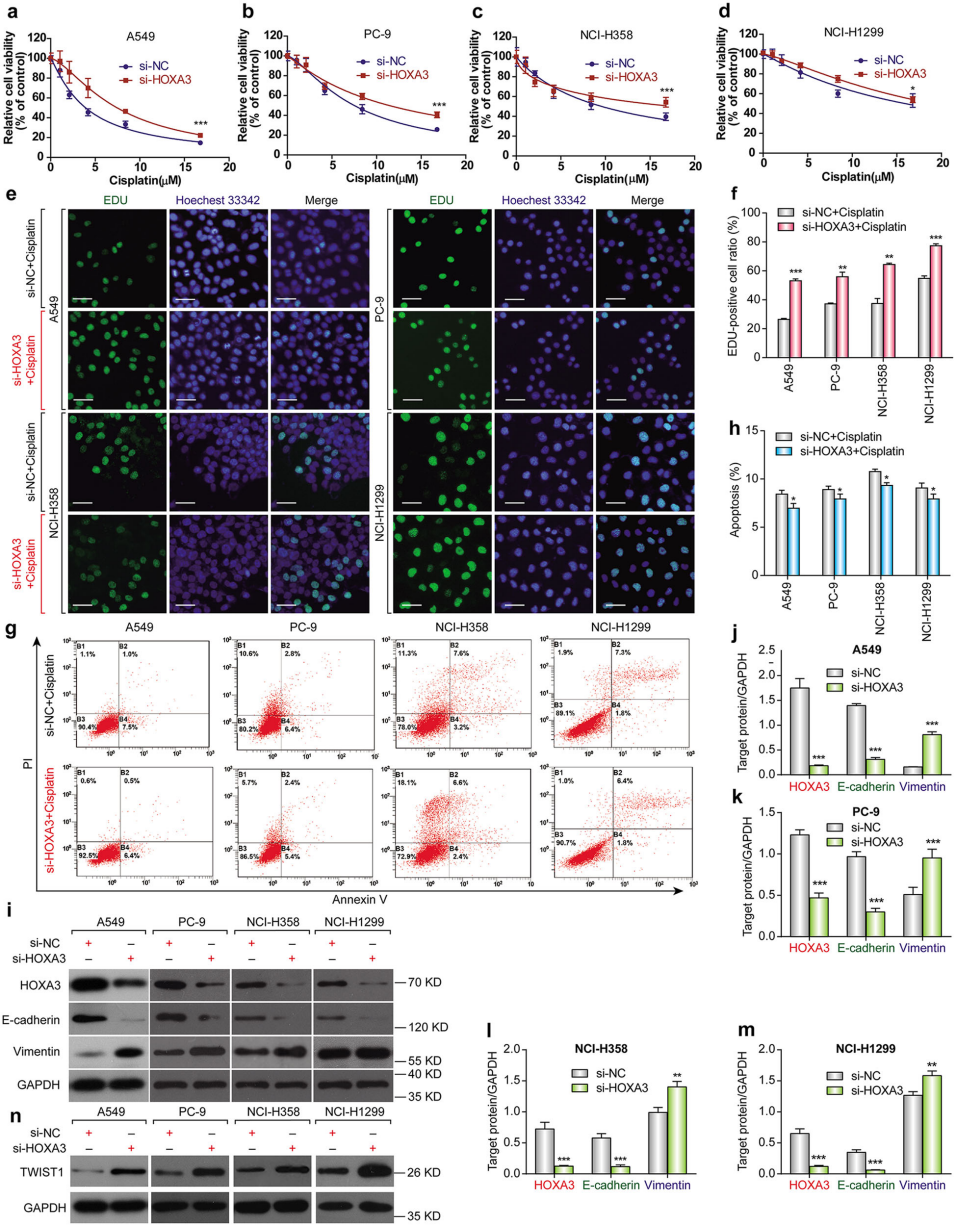
HOXA3 knockdown increased cisplatin resistance through EMT. **a**–**d** CCK-8 assay of the viability of NSCLC cell lines following cisplatin treatment (0, 1.049, 2.097, 4.195, 8.389, 16.779 μM) under HOXA3 suppression for 48 h. **e**–**f** EDU assay of the proliferation of NSCLC cell lines under the IC_50_ of cisplatin under HOXA3 suppression for 48 h. All scale bars indicate 50 μm (Green, EDU staining of dividing cells; blue, Hoechest 33342 staining of nuclei). **g**, **h** Flow cytometry detection of the apoptosis rate of NSCLC cell lines under the IC_50_ of cisplatin under HOXA3 suppression for 48 h. **i**–**m** Western blotting of HOXA3, E-cadherin, and Vimentin levels following HOXA3 suppression for 48 h. **n** Western blotting of Twist1 levels following HOXA3 suppression for 48 h. (*p < 0.05, **p < 0.01, ***p < 0.001)

HOXA-AS3 knockdown decreases cisplatin resistance and increases HOXA3 expression; therefore, we investigated whether HOXA3 depletion would affect cisplatin resistance. CCK-8 assays showed that HOXA3 knockdown in NSCLC cells resulted in a significant increase in viability in response to cisplatin compared with that in the control cells (Fig. 5a–d). The IC_50_ values of cisplatin also increased significantly in the HOXA3 knockdown cells compared with those in the controls (Supplementary Table 1.4). EDU staining assays confirmed that knockdown of HOXA3 increased NSCLC cell viability following cisplatin treatment compared with that in the control cells (Fig. 5e, f). Furthermore, flow cytometry showed that HOXA3 knockdown decreased apoptosis following cisplatin treatment (Fig. 5g, h). HOXA3 knockdown in NSCLC cell lines increased their resistance to cisplatin; therefore, we decided to investigate the underlying mechanism of this effect. Emerging evidence suggests a molecular and phenotypic association between EMT and drug resistance in several cancers. Thus, we investigated whether knockdown of HOXA3 influenced EMT markers using western blotting analysis. In all four NSCLC cell lines, HOXA3 knockdown resulted in reduced expression of E-cadherin and increased expression of Vimentin and Twist1 (Fig. 5i–n), indicating that HOXA3 knockdown induced EMT in the NSCLC cell lines.

In order to determine the effect of EMT on HOXA3-mediated cisplatin sensitization, we transfected NCSLC cells with siRNA negative, Twist1 siRNA or Twist1 siRNA + HOXA3 siRNA, respectively. CCK-8 assays showed that Twist1 knockdown enhanced cisplatin sensitivity in NSCLC cells compared with that in the controls. In contrast, knockdown of HOXA3 did not reverse the effect of Twist1 siRNA (Fig. S2a). Moreover, the result of western blotting showed that there was no significant difference in EMT-related protein level between Twist1 siRNA group and Twist1 siRNA + HOXA3 siRNA group (Fig. S2b). These results showed that HOXA3 regulated cisplatin sensitivity via EMT.

### HOXA-AS3 knockdown decreases cisplatin resistance by upregulating HOXA3

**Fig. 6:**
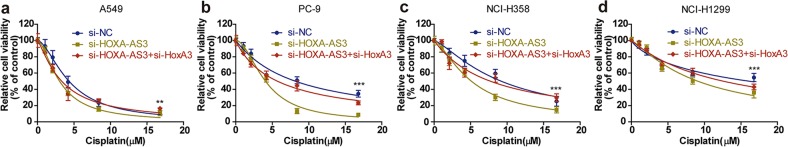
HOXA-AS3 deletion decreased cisplatin resistance via upregulating HOXA3. **a**–**d** CCK-8 assay of the viability of NSCLC cell lines following cisplatin treatment (0, 1.049, 2.097, 4.195, 8.389, 16.779 μM) under HOXA-AS3 + HOXA3, or HOXA-AS3 only suppression for 48 h. (*p < 0.05, **p < 0.01, ***p < 0.001)

We next examined whether HOXA3 plays an important role in HOXA-AS3-induced cisplatin resistance using double-knockdown experiments. As mentioned previously, CCK-8 assays showed that HOXA-AS3 knockdown in NSCLC cells resulted in a significant reduction in viability in response to cisplatin compared with that in the controls. In contrast, knockdown of both HOXA-AS3 and HOXA3 restored viability to control levels in response to cisplatin (Fig. 6a–d). The IC_50_ values of cisplatin in the double-knockdown NSCLC cells were also similar to those in the control cells (Supplementary Table 1.5). Thus, HOXA3 knockdown fully reversed the increase in cisplatin resistance conferred by HOXA-AS3 knockdown. These results suggest that HOXA-AS3 confers cisplatin resistance by upregulating HOXA3.

### Antitumor effects of HOXA-AS3 knockdown on xenograft tumors in mice

**Fig. 7:**
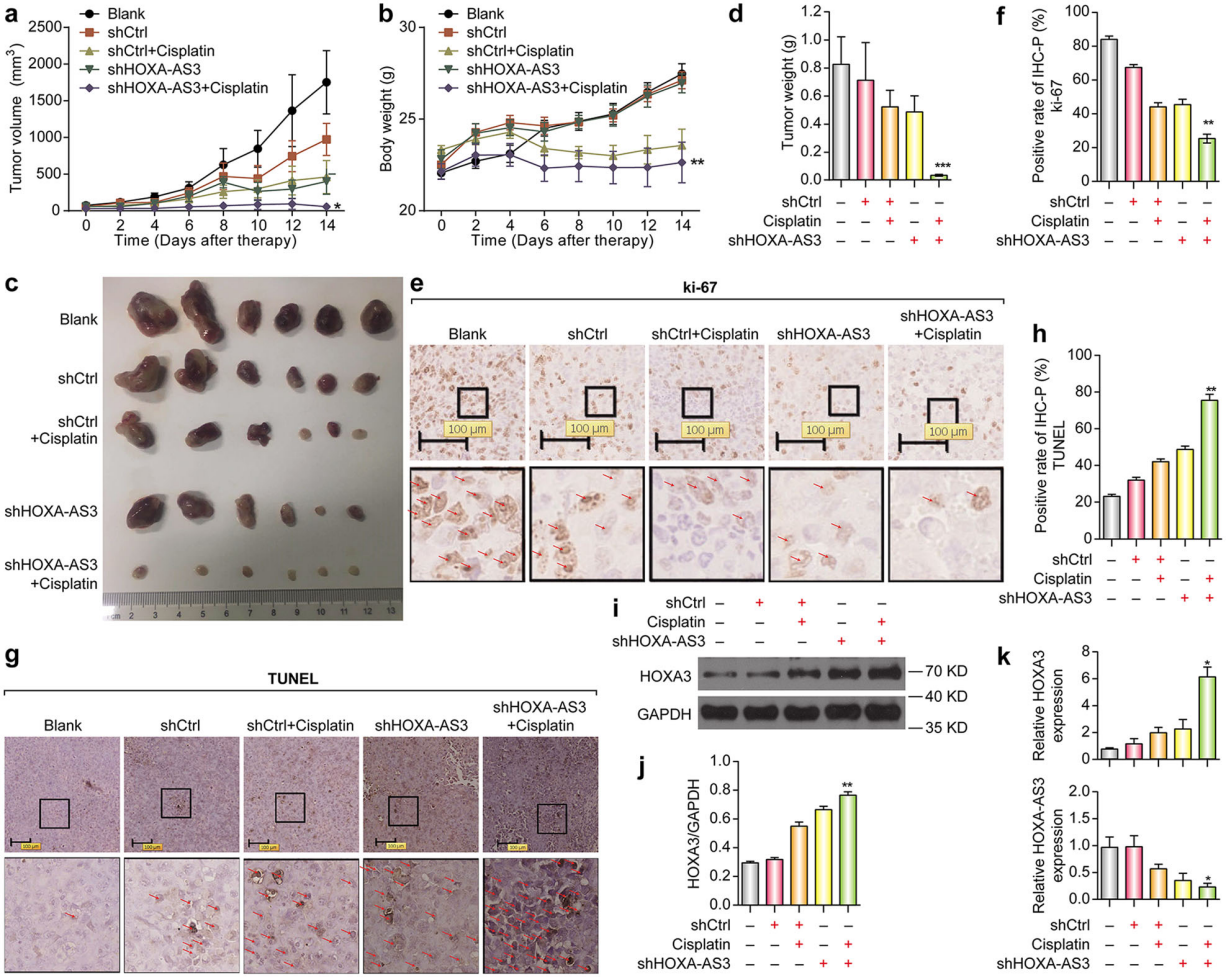
Antitumor effects of HOXA-AS3 inhibition on tumor xenograft mice. **a** Growth curve of tumor volumes and **b** Growth curve of body weight calculated every 2 days after inoculation. **c** Tumor size after the tumors was harvested. **d** Tumor weight after the tumors was harvested. **e**–**f** Representative images of immunohistochemical detection of the proliferation index Ki-67 in vivo. **g**, **h** Representative images of immunohistochemical detection of the apoptosis index TUNEL in vivo. **i**, **j** Western blotting detection of HOXA3 in vivo. **k** qPCR detection of the mRNA expression levels of *HOXA3* and HOXA-AS3 in vivo. (* vs cisplatin, *p < 0.05, **p < 0.01, ***p < 0.001)

To further investigate the impact of HOXA-AS3 inhibition on tumor progression in vivo, we performed a xenograft experiment in nude mice. NCI-H1299 cells were transplanted subcutaneously into the nude mice. After the tumor volume reached 100–200 mm^3^, mice were treated with shCtrl, a combination of shCtrl and cisplatin, shHOXA-AS3, or a combination of shHOXA-AS3 and cisplatin. Tumor growth was monitored for 14 days. The tumor volume gradually increased in the control mice in a time-dependent manner. Similarly, treatment with shCtrl, shCtrl+cisplatin, or shHOXA-AS3 resulted in increases in tumor volumes and body weight. In contrast, under treatment with shHOXA-AS3+cisplatin, increases in tumor volumes and body weight were minimal (Fig. 7a, b). Moreover, the tumor size and weight were markedly decreased in the shHOXA-AS3+cisplatin group compared to those in the other groups (Fig. 7c, d). Furthermore, IHC analysis of the tumors showed that the proportion of cells positive for the cell proliferation indicator Ki67 was reduced in the shHOXA-AS3+cisplatin group (Fig. 7e, f). In contrast, the proportion of cells positive for the cell apoptosis indicator, TUNEL, was elevated following treatment with shHOXA-AS3+cisplatin (Fig. 7g, h). H&E-stained sections of the liver (Fig. S3a) and kidney (Fig. S3b) showed no metastasis in any of the nude mice. The H&E assay results also indicated that the tumors treated with shHOXA-AS3+cisplatin exhibited no significant alterations in cell shape or karyopyknosis, as compared with those in the other groups (Fig. S3c). These results in a well-established xenograft mouse model strongly suggested that inhibition of HOXA-AS3 augments the efficacy of cisplatin to treat lung cancer.

To further investigate the relationship between HOXA-AS3 and HOXA3, we performed western blotting and qPCR to detect the expression of HOXA-AS3 or HOXA3 in tumor tissues from nude mice. The protein expression of HOXA3 in the shHOXA-AS3 group was upregulated compared with that in the shCtrl group; whereas HOXA3 was upregulated when cisplatin was added in the presence of shHOXA-AS3 (Fig. 7i, j). The mRNA level of HOXA3 was upregulated following treatment with shHOXA-AS3+cisplatin compared with that in the cispaltin group (Fig. 7k). Thus, HOXA-AS3 knockdown enhanced cisplatin efficacyand upregulated HOXA3 in vivo, confirming the in vitro results. We then examined HOXA-AS3 expression levels and found them to be higher in the shCtrl group than in both the shHOXA-AS3 and shHOXA-AS3+cisplatin groups (Fig. 7k). This suggested that the HOXA-AS3 expression levels in tumors in vivo were moderately inhibited by cisplatin treatment, which was not found in the cell lines (Fig. 1e). This discrepancy may be because in cell line results, HOXA-AS3 was detected only in the cells that survived cisplatin treatment, whereas in the animal model, HOXA-AS3 RNA levels in both surviving and damaged cells was examined, as whole tissue was extracted. Taken together, our findings indicated that HOXA-AS3 inhibition augments the efficacy of cisplatin by upregulating HOXA3 in vivo.

## Discussion

Recently, many studies have indicated that the aberrant expression of lncRNAs is responsible for drug resistance, which is a substantial obstacle to effective cancer therapy. Targeting lncRNAs for cancer intervention is a novel and promising therapeutic approach^13^. For example, DTA-H19, a double-stranded DNA plasmid, carries the gene for the diphtheria toxin A subunit, which causes cell death by immediately ending gene translation and the H19 promoter limits its expression to tumor cells^14^. Promising anticancer effects of DTA-H19 have been demonstrated in mouse models of lung cancer, as well as in colon, gastric, bladder, and ovarian cancers. Surprisingly, intratumoral injection of DTA-H19 in patients has been shown to reducethe tumor volume in bladder or pancreatic cancer^15–21^. To develop additional therapeutic approaches based on lncRNAs, it is necessary to further investigate the role of lncRNAs in drug resistance.

HOXA-AS3 is located on chromosome 7p15.2, which contains the HOXA gene cluster. The HOX gene family consists of 39 genes and plays a major role in tissue development and cancers^22,23^. In previous studies, HOXA-AS3 was found to be upregulated in glioma tissues and cell lines, and high expression of HOXA-AS3 is associated with poor prognosis in patients with glioma. HOXA-AS3 acts as an oncogene in glioma by increasing cell proliferation, inhibiting apoptosis, and promoting cell migration^24^. Studies have determined that HOXA-AS3 acts as an epigenetic switch that determines the lineage specification of mesenchymal stem cells, interacts with EZH2, and is required for H3K27me3 deposition on the key osteogenic transcription factor gene RUNX2^25^. In the present study, the results from in vitro and in vivo models suggested that HOXA-AS3conferred cisplatin resistance. Thus, our study provided the first characterization of the role of HOXA-AS3 in mediating drug resistance, establishing HOXA-AS3 as a candidate drug target to develop more efficient cisplatin-based treatment for NSCLC. Moreover, the pattern of HOXA-AS3 expression in NSCLC cells appeared to correlate with resistance to cisplatin, indicating that HOXA-AS3 might be a useful biomarker to predict cisplatin resistance in these cancers.

Combination chemotherapy has become the norm in lung cancer treatment. Although we focused on cisplatin resistance in the present study, we expect to uncover roles of HOXA-AS3 in mediating the drug response of lung cancer cells to other chemotherapeutic agents, which will enhance our overall understanding of the roles of lncRNAs in cancer drug resistance. In turn, a better understanding of the molecular mechanisms by which HOXA-AS3 affects drug resistance will promote the development of more effective cancer treatments. Notably, overexpression of HOXA-AS3 did not affect drug resistance. There is no clear explanation for this; however, it could be causedby “cis-effects”, meaning that the RNA has to be delivered to specific chromatin regions to function. Also, for treatment, HOXA-AS3 overexpression is not required; inhibitingHOXA-AS3 would be sufficient.

LncRNAs regulate target genes through various mechanisms, including chromatin remodeling, transcriptional control, posttranscriptional processing, protein functioning and localization, and intercellular signaling^26–28^. Our study is the first to show that HOXA-AS3 knockdown increased the mRNA and protein levels of HOXA3, and that HOXA-AS3 interacts with both the HOXA3 mRNA and protein. HOXA3 belongs to the homeobox family of genes, which encode highly conserved transcription factors that are important for physiological functions, such as early embryonic development, and thymus and parathyroid differentiation^29,30^. Moreover, HOXA3 has various functions in the immune system and nervous system, such as regulation of macrophage activation, promotion of the differentiation of hematopoietic precursor cells into myeloid cells, and prevention of aberrant neuronal identity and behavior^31–33^. In tumors, HOXA3 is reported to promote human colon cancer formation by regulating the EGFR/Ras/Raf/MEK/ERK signaling pathway^34^. In addition, DNA hypermethylation of *HOXA3* has been identified as a biomarker for lung adenocarcinoma^35^. However, the detailed functions and mechanisms of HOXA3 in lung cancer have not been studied. In the current study, HOXA3 was identified as a key downstream effector of HOXA-AS3, which confers cisplatin resistance in vitro and in vivo. Indeed, *HOXA3* knockdown significantly increased cisplatin resistance.

EMT has been shown to play an important role in drug resistance in many tumors, including breast cancer, lung cancer, colon cancer, and pancreatic cancer^36–38^. Therefore, EMT has become a prime target of interest in anticancer therapy^39,40^. HOXA3 induces migration and angiogenesis of endothelial and epithelial cells in response to injury^41^. Furthermore, in a mouse model of idiopathic pulmonary fibrosis, HOXA3 was reported as an important modulator of EMT^42^. In the present study, suppressing HOXA3 downregulated E-cadherin and upregulated Vimentin levels, suggesting that *HOXA3* knockdown increased drug resistance in NSCLC cells by inducing EMT. This implied that EMT plays an important role in HOXA-AS3-mediated cisplatin resistance. However, further study is warrantedto fully dissect the molecular mechanisms by which HOXA3 regulates the expression of EMT genes.

Several additional investigations are needed before a full understanding of the mechanism of HOXA-AS3-mediated drug resistance is achieved. First, correlations between HOXA-AS3/HOXA3 expression and clinical outcomes in NSCLC patients should be investigated. Moreover, additional studies are needed to investigate other mechanisms and signaling pathways that may be involved in the mechanism by which HOXA-AS3 confers cisplatin resistance to NSCLC cells. In addition, the mechanism by which HOXA3 affects the process of EMT should be studied in greater detail. Lastly, investigations into the details of how HOXA-AS3 affects the response of lung cancer cells to other chemotherapeutic agents should be performed to provide a deeper understanding of the underlying molecular details of HOXA-AS3-mediated drug resistance.

In conclusion, HOXA-AS3 confers cisplatin resistance. Additionally, HOXA-AS3 knockdown reduced drug resistance by upregulating HOXA3 expression, which increases cisplatin resistance and induces EMT. Taken together, our findings suggest that research into HOXA-AS3 may provide a better understanding of the mechanisms of drug resistance and enable the development of novel and efficient strategies to treat NSCLC.

## Materials and methods

### Cell lines and cell culture

Human lung cancer cell lines A549, NCI-H1299, NCI-H358, and PC-9 were obtained from the American Type Culture Collection (ATCC; Manassas, VA, USA) or the Cell Bank of Shanghai Institutes for Biological Sciences, the Chinese Academy of Sciences (Shanghai, China). Cells were cultured as recommended by relevant cell bank.

### Cell counting Kit-8 assay

Chemosensitivity was determined using a Cell Counting Kit-8 assay (CCK-8; Dojindo Laboratories, Kumamoto, Japan). Briefly, 5 × 10^3^ cells in 100 μl of medium were dispensed into a 96-well plate. After overnight incubation, the cells were exposed to cisplatin for 48 h. Then, the CCK-8 reagent was added to the wells and incubated for 1 h. Finally, the absorbance of the sample in each well was detected at 450 nm.

### Western blotting, hematoxylin and eosin staining, and immunohistochemistry analysis

After the cells had completely adhered to the wells, the culture medium was replaced with medium containing 10% fetal bovine serum (FBS; GIBCO, Grand Island, USA), and the half maximal inhibitory concentration (IC50) of cisplatin was determined. Western blotting was performed as described previously^43^. Cell lysates were generated, and total protein was separated by standard SDS-PAGE, followed by transfer to polyvinylidene fluoride (PVDF) membranes. The membranes were then washed and blocked before incubation with the primary antibodies recognizing the following proteins: HOXA3 (SC-374237, Santa Cruz Biotechnology, Santa Cruz, CA, USA), E-cadherin (#3195, Cell Signaling Technology, Danvers, MA, USA), Vimentin (#5741, Cell Signaling Technology), Twist1 (#46702, Cell Signaling Technology) and GAPDH (#2118, Cell Signaling Technology). This was followed by incubation with horseradish peroxidase (HRP)-conjugated secondary antibodies (#7074 and #7076, Cell Signaling Technology). Reactions were detected using enhanced chemiluminescence assays. GADPH was used as a control.

Hematoxylin and eosin (H&E) staining, immunohostochemistry (IHC), and their subsequent evaluation were performed as described previously^43^.

### RNA preparation and quantitative real-time reverse-transcription PCR

All procedures were performed according to the manufacturer’s instructions. Total RNA was extracted using an Ultrapure RNA kit (CWbio, Co., Ltd., Beijing, China). RNA was reverse-transcribed into cDNA using iScript cDNA Synthesis kits (Bio-Rad Laboratories, Hercules, CA, USA). Quantitative real-time PCR (qPCR) was performed using the SYBR-Green PCR kit (Applied Biosystems, Foster City, CA, USA). GADPH was used as a loading control. Specific primers for the amplification of the target genes and GAPDH are listed in Supplementary Table 2.1.

### Small interfering RNA and plasmid vector transfection

HOXA-AS3-overexpressing cells were generated according to a standard protocol. Briefly, the full-length HOXA-AS3 cDNA was cloned into vector pCDNA3.1, and cells were transfected using Lipofectamine 3000 (L3000015, Thermo Fisher Scientific, Waltham, MA, USA). Stable clones were obtained by selection with G418. All constructs were confirmed by DNA sequencing. Small interfering RNAs (siRNAs) targeting HOXA-AS3 (si-HOXA-AS3-651, si-HOXA-AS3-728, si-HOXA-AS3-3507), HOXA3 (#sc-38675, Santa Cruz Biotechnology) or negative control (NC) siRNAs were transfected into NSCLC cells using Lipofectamine 3000. All siRNA sequences are shown in Supplementary Table 2.2.

### Ethynyldeoxyuridine assay

Proliferating NSCLC cell lines were investigated using a Click-iT Ethynyldeoxyuridine (EDU) Imaging Kit (Invitrogen, Carlsbad, CA, USA) according to the manufacturer’s protocol. Briefly, cells were incubated with the IC_50_ of cisplatin for 48 h and then with 10 µM EDU for 2 h, before fixation, permeabilization, and staining for EDU. Nuclei were counterstained with 5 µg/ml Hoechst 33342 (Invitrogen) for 30 min.

### Immunofluorescence

NSCLC cells were seeded into at 3 × 10^3^ cells/well in 48-well plates, fixed with 4% formaldehyde for 15 min, washed with PBS, and treated with 5% bovine serum albumin (BSA) for 30 min at room temperature. Then the cells were then incubated with primary antibodies (anti-E-cadherin, anti-Vimentin, dilution 1:200; Cell Signaling Technology) at 4 °C overnight. The cells were incubated with fluorescein isothiocyanate (FITC)-conjugated secondary antibody (Abcam, Cambridge, MA, USA) at 4 °C for 2 h. Nuclear staining was performed using 2-(4-amidinophenyl)-1H-indole-6-carboxamidine (DAPI; Sigma, St. Louis, MO, USA) at room temperature for 5 min. After washing with PBS, the NSCLC cells were observed using an inverted fluorescence microscope (Olympus, Tokyo, Japan).

### Flow cytometry analysis

Apoptosis analysis was performed using an FITC Annexin V Apoptosis Detection Kit I (BD Biosciences,San Jose, CA, USA). Briefly, cells were harvested using trypsin, washed twice with ice-cold PBS, and resuspended in 1 × Binding Buffer at a concentration of 1 × 10^6^ cells/ml. Then, 100 µl of the solution was transferred into a 5-ml culture tube, and 5 µl of propidium iodide (PI) and 5 µl of FITC Annexin V were added. After incubation for 15 min at 25 °C in the dark, 400 µl of 1 × Binding Buffer was added to each tube, and the stained cells were analyzed using a FACSC alibur Flow Cytometer (BD Biosciences).

### RNA pull-down assay and RNA immunoprecipitation

We performed RNA pull-down assays using the Pierce™ Magnetic RNA-Protein Pull-Down Kit (#20164, Thermo Fisher Scientific). Briefly, RNA was first bound to the beads to orient the RNA for protein binding. The RNA-bound beads were then equilibrated in Protein-RNA Binding Buffer before the addition of the protein lysate. The beads were then washed by adding the appropriate buffer, vortexing, and separating on a magnetic stand. The specificity of the RNA pull-down was assessed using western blotting.

For RNA immunoprecipitation, supernatants were incubated with anti-HOXA3 or control IgG antibodies and then with Protein A/G beads (#20421, Thermo Fisher Scientific). Total RNA that was retained on the antibody-beads was extracted, and HOXA-AS3 enrichment was determined using qPCR.

### Mass spectrometry

Protein (0.5 μL) obtained by centrifugation was uniformly coated on the 96-well target plate and dried at room temperature. Each well was then overlaid with 0.5 μL α-cyano-4-hydroxycinnamic acid (Fluka) saturated with a 50:47.5:2.5 mixture of acetonitrile:water:trifluoroacetic acid (Sigma, Ameican). After the sample was dried, the target plate is placed into the Microflex LT MALDI-TOF MS instrument (Bruker Daltonics, Germany) for detection. The parameters of the instrument were set in the positive linear operation mode, the mass range collected by MALDI-TOF MS was 2,000~20,000, the acceleration voltage was 20 kV, the extraction voltage was 18.6 kV, the focusing voltage was 6.5 kV, and the extraction delay time was 150 ns. Calibration was performed with Protein standard I. Mass spectra were analyzed by Flex Control 3.0.

### Lentivirus transfection and antitumor effects on tumor xenografts in nude mice

Lentivirus particles expressing nonsense short hairpin RNA (shRNA) (shCtrl) or shRNA targeting HOXA-AS3 (shHOXA-AS3) were generated by transfecting 293T cells with shCtrl or shHOXA-AS3 plasmids, together with packaging plasmids. The sequences of the shRNAs are shown in Supplementary Table 2.2.

The animal studies were approved by the Institutional Animal Care and Use Committee of Zhejiang University, Hangzhou, China. Male 4-5-week-old BALB/c nude mice were used in this study. NCI-H1299 cells (2 × 10^6^) were suspended in 100 µl of serum-free medium and inoculated into the mice subcutaneously. Approximately 2 weeks later, when the average tumor volume reached 100–120 mm^3^, the mice were randomly divided into groups (*n* = 8). Then, 50 µl of shCtrl or shHOXA-AS3 (1 × 10^8^ transducing units (TU)/ml)were injected into each tumor on day 0 and day 8. Cisplatin (2 mg/kg mouse body weight) was administered daily for 2 weeks by tail vein injection. Tumor sizes were measured using a Vernier caliper each week thereafter, and tumor volumes (mm^3^) were calculated as length × width^2^/2.

### Statistical analysis

Statistical analyses were performed using GraphPad Prism software version 5.0 (GraphPad Software, Inc., La Jolla, CA, USA) and SPSS version 19.0 (IBM Corp., Armonk, NY, USA). Statistical analysis of group differences was performed using the chi-squared test or Student’s *t*-test. The mean Standard Deviation (SD) of three independent experiments was determined. *P*-values < 0.05 were considered statistically significant.

## Supplementary information

Supplementary figure legends

Supplementary Figure 1

Supplementary Figure 2

Supplementary Figure 3

Supplementary Table 1

Supplementary Table 2
