# Supplementary material for: LncRNA HOXA-AS3 confers cisplatin resistance by interacting with HOXA3 in non-small-cell lung carcinoma cells
Source: Oncogenesis. 2019 Oct 15;8(11):60. doi: 10.1038/s41389-019-0170-y (PMC6794325; doi:10.1038/s41389-019-0170-y)
Supplement: Supplementary file 1 — Supplementary figure legends [file 41389_2019_170_MOESM1_ESM.docx]

**Supplementary files**

**Figure S1**. Upregulation of HOXA-AS3 expression does not affect cisplatin resistance.

(a-d) CCK-8 assay detection of the viability of NSCLC cell lines following cisplatin treatment under HOXA-AS3 upregulation. (e) qPCR analyses detection of the mRNA expression of HOXA3 following HOXA-AS3 upregulation. (*p < 0.05, **p < 0.01, ***p < 0.001)

**Figure S2.** The effect of EMT on HOXA3-mediated cisplatin sensitization

(a) CCK-8 assay detection of the viability of NSCLC cell lines following cisplatin treatment after transfection of si-TWIST1 or si-TWIST1+si-HOXA3.(b)Western blooting detection of the expression of E-cadherin,Vimentin and TWIST1 after transfection of si-TWIST1 or si-TWIST1+si-HOXA3.

**Figure S3**. Representative images of H&E from nude mice. (a) Representative images of H&E of liver from nude mice. (b) Representative images of H&E of kidney from nude mice. (c) Representative images of H&E of cell morphology from tumor.
