# Supplementary figures and images for: LncRNA HOXA-AS3 confers cisplatin resistance by interacting with HOXA3 in non-small-cell lung carcinoma cells
Source: Oncogenesis. 2019 Oct 15;8(11):60. doi: 10.1038/s41389-019-0170-y (PMC6794325; doi:10.1038/s41389-019-0170-y)

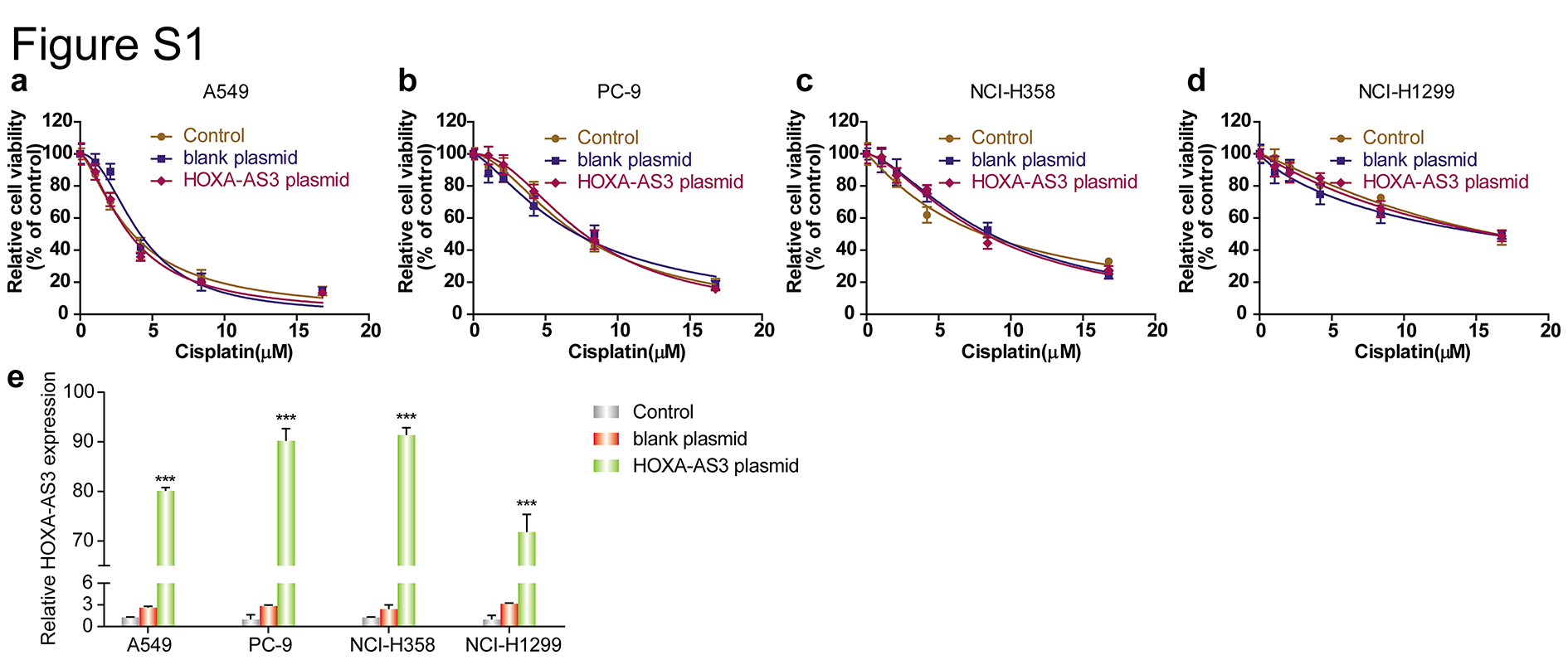

Supplement: Supplementary file 2 — Supplementary Figure 1 [file 41389_2019_170_MOESM2_ESM.tif]

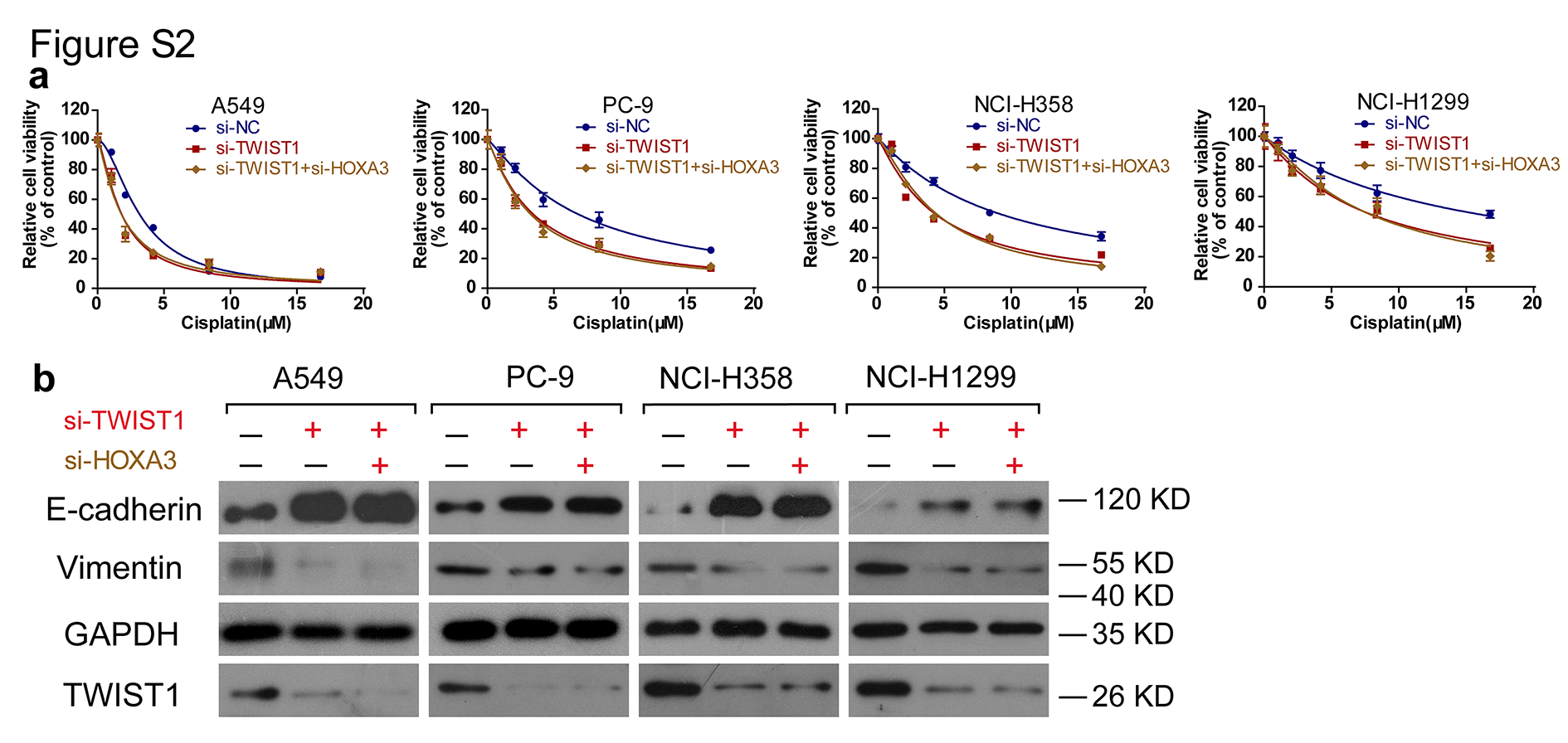

Supplement: Supplementary file 3 — Supplementary Figure 2 [file 41389_2019_170_MOESM3_ESM.tif]

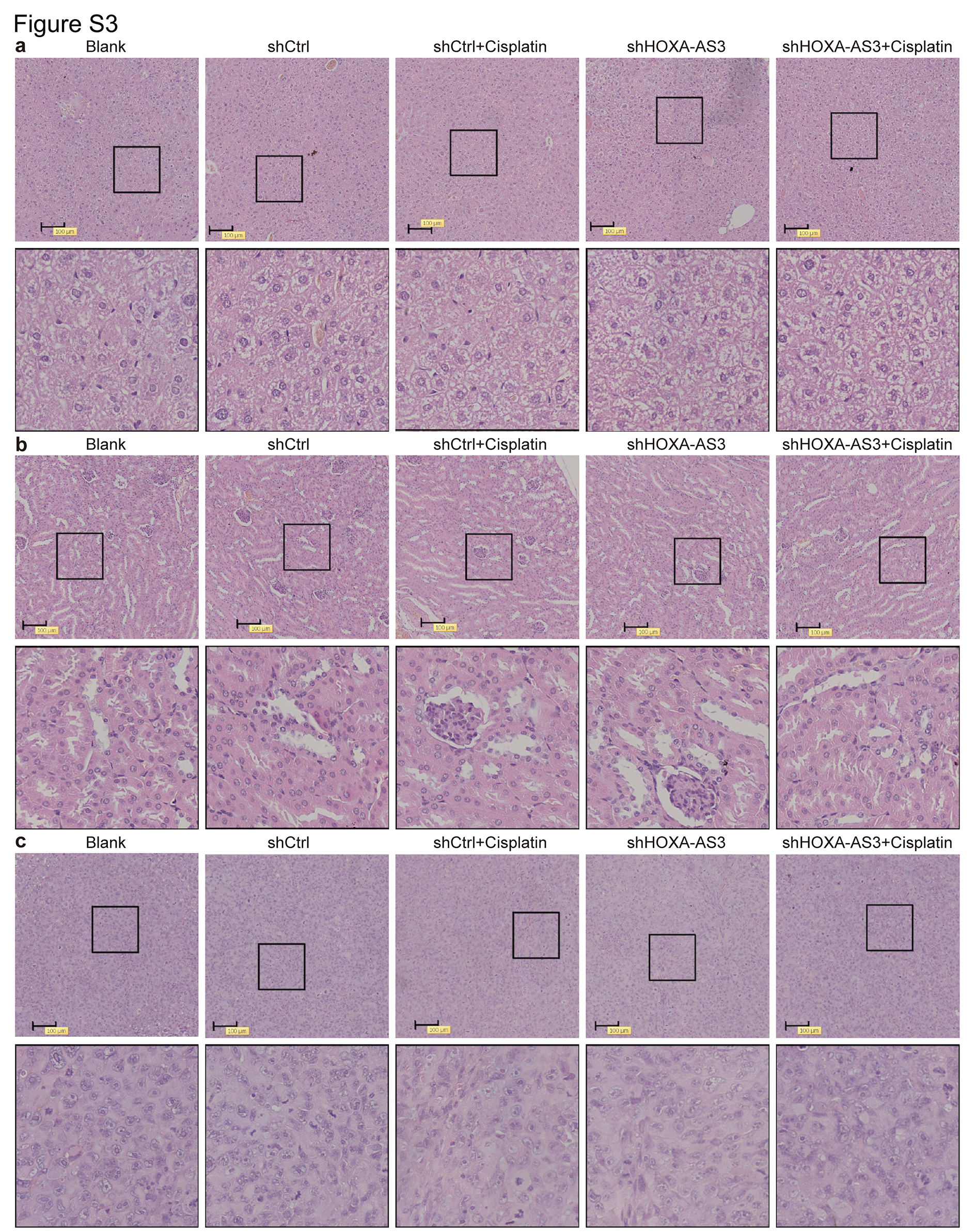

Supplement: Supplementary file 4 — Supplementary Figure 3 [file 41389_2019_170_MOESM4_ESM.tif]
