# Supplementary material for: LncRNA HOXA-AS3 confers cisplatin resistance by interacting with HOXA3 in non-small-cell lung carcinoma cells
Source: Oncogenesis. 2019 Oct 15;8(11):60. doi: 10.1038/s41389-019-0170-y (PMC6794325; doi:10.1038/s41389-019-0170-y)
Supplement: Supplementary file 5 — Supplementary Table 1 [file 41389_2019_170_MOESM5_ESM.doc]

**Supplement Tables**

1.IC50 of NSCLC cell lines for Cisplatin.

| IC_50_ (μM) | | | |
| --- | --- | --- | --- |
| A549 | PC-9 | NCI-H358 | NCI-H1299 |
| 3.027(2.730 to 3.323 ) | 7.205(8.087 to 8.735) | 9.51(8.735 to 10.208) | 4.760(14.923to17.023) |

IC_50_ values show Cisplatin concentration [μM. mean (95% confidence intervals)].

2.IC50 of NSCLC cell lines for Cisplatin after HOXA-AS3 knockdown.

| cell lines | IC_50_ (μM) | | | |
| --- | --- | --- | --- | --- |
|  | si-NC | si-HOXA-AS3-651 | si-HOXA-AS3-728 | si-HOXA-AS3-3507 |
| A549 | 4.453(4.107 to 4.799) | 3.172(2.942 to3.403) | 2.709(2.491 to 2.928) | 3.288(2.983 to 3.591) |
| PC-9 | 8.195(7.584 to 8.802) | 5.436(4.594 to 6.279) | 3.970(3.527 to 4.409) | 5.765(5.262 to 6.265) |
| NCI-H358 | 9.695(9.074 to 10.315) | 7.131(6.372to 7.889) | 4.534(4.154 to 4.909) | 6.352(5.728 to 6.977) |
| NCI-H1299 | 17.500(14.688 to 20.332) | 8.678(8.111 to 9.245) | 8.342(7.758 to 8.809) | 7.758(7.305 to 8.215) |

IC_50_ values show Cisplatin concentration [μM. mean (95% confidence intervals)].

3. IC50 of NSCLC cell lines for Cisplatin after HOXA-AS3 overexpression.

| cell lines | IC_50_ (μM) | | |
| --- | --- | --- | --- |
|  | Control | blank plasmid | HOXA-AS3 plasmid |
| A549 | 3.641(3.379 to3.899) | 4.168(4.406 to 4.537) | 3.426(3.170 to 3.681) |
| PC-9 | 7.168(6.711 to 7.805) | 7.201(6.567 to 7.832) | 7.668(7.258 to 8.074) |
| NCI-H358 | 8.087(7.342 to 8.836) | 8.587(7.933 to 9.242) | 8.195(7.584 to 8.802) |
| NCI-H1299 | 16.423(14.641 to18.205) | 15.943(12.953 to 18.930) | 16.389(14.097 to 18.409) |

IC_50_ values show Cisplatin concentration [μM. mean (95% confidence intervals)].

4.IC50 of NSCLC cell lines for Cisplatin after HOXA3 knockdown.

| cell lines | IC_50_ (μM) | |
| --- | --- | --- |
|  | si-NC | si- HOXA3 |
| A549 | 3.933 (3.597 to 4.265)  7.497 6.909 to 8.081)  9.587 (8.322 to 10.839)  15.966 (13.305 to 18.624) | 7.470 (6.695 to8.248)  11.393 (10.124 to 12.658)  17.617 (11.819 to23.416)  18/990(17.517to 20.463) |
| PC-9 |  |  |
| NCI-H358 |  |  |
| NCI-H1299 |  |  |

IC_50_ values show Cisplatin concentration [μM. mean (95% confidence intervals)].

5.IC50 of NSCLC cell lines for Cisplatin after HOXA-AS3 and HOXA3 knockdown.

| cell lines | IC_50_ (μM) | | |
| --- | --- | --- | --- |
|  | si-NC | si-HOXA-AS3 | si-HOXA-AS3+ si-HOXA3 |
| A549 | 4.325 (3.973 to 4.681) | 3.029(2.866 to3.191) | 3.319 (2.975 to3.664) |
| PC-9 | 8.174 7.245 to 9.101) | 4.074 (3.799to 4.349) | 5.715 (5.191 to 6.242) |
| NCI-H358 | 9.205 (8.218 to 10.188) | 5.121 (4.748 to 5.490) | 7.725 (6.549 to 8.856) |
| NCI-H1299 | 16.161(12.795 to 19.530) | 9.097 (8.195 to10.000) | 12.604 (11.503 to 13.691) |

IC_50_ values show Cisplatin concentration [μM. mean (95% confidence intervals)].
