# Supplementary material for: LncRNA HOXA-AS3 confers cisplatin resistance by interacting with HOXA3 in non-small-cell lung carcinoma cells
Source: Oncogenesis. 2019 Oct 15;8(11):60. doi: 10.1038/s41389-019-0170-y (PMC6794325; doi:10.1038/s41389-019-0170-y)
Supplement: Supplementary file 6 — Supplementary Table 2 [file 41389_2019_170_MOESM6_ESM.docx]

Table 2

1. Sequences of gene-specific primers used for real-time RT-PCR.

| Gene | Primer Sequence Forward (5’-3’) | Primer Sequence Reverse (5’-3’) |
| --- | --- | --- |
| HOXA-AS3 | CACCTCTCTCATCGAAAAACCG | GCACCAGGAAAGAGGACAATTC |
| HOXA3 | CAGCTCATGAAACGGTCTGC | GAGCTGTCGTAGTAGGTCGC |
| ATCB | TGGCACCCAGCACAATGAAACT | CTAAGTCATAGTCCGCCTAGAAGCA |

2. Sequences of siRNA and shRNA.

| Gene | sence | antisence | |  |
| --- | --- | --- | --- | --- |
| si-HOXA-AS3-651 | UCUAUUCUCGCAAGGGAAATT | | UUUCCCUUGCGAGAAUAGATT | |
| si-HOXA-AS3-728/shRNA | GGGCCGAACAACUCAUAAATT | | UUUAUGAGUUGUUCGGCCCTT | |
| si-HOXA-AS3-3507 | GCACAGAAUCUCAACUUUATT | | UAAAGUUGAGAUUCUGUGCTT | |
| Twist1-homo-1575 | GGUGUCUAAAUGCAUUCAUTT | | AUGAAUGCAUUUAGACACCTT | |
| Twist1-homo-810 | GGUACAUCGACUUCCUCUATT | | UAGAGGAAGUCGAUGUACCTT | |
| Twist1-homo-780 | GCAAGAUUCAGACCCUCAATT | | UUGAGGGUCUGAAUCUUGCTT | |
